# A study on the impact of need satisfaction on perceived academic performance among arts major undergraduate students in China—focusing on the mediating effect of achievement goals orientations and the moderating effect of perceived social support

**DOI:** 10.3389/fpsyg.2026.1692993

**Published:** 2026-03-17

**Authors:** Xinzhou Gao, Miao Chen, Yuntao Li

**Affiliations:** College of Music and Dance, Weifang University, Weifang, Shandong, China

**Keywords:** achievement goal orientations, art major undergraduates, perceived academic performance, perceived social support, self-determination theory

## Abstract

Drawing on Self-Determination Theory (SDT), this study examines the psychological mechanisms underlying perceived academic performance among Chinese undergraduate students majoring in the arts. Specifically, it investigates the direct effects of basic psychological need satisfaction—autonomy, competence, and relatedness—on perceived academic performance, as well as the mediating role of achievement goal orientations and the moderating role of perceived social support. Using a cross-sectional survey design, data were collected from art major undergraduates across multiple universities in China and analyzed using structural equation modeling. The results indicate that all three psychological needs exert significant positive direct effects on perceived academic performance. Achievement goal orientations partially mediate the effects of competence and relatedness, but not autonomy, suggesting distinct motivational pathways across needs. In addition, perceived social support negatively moderates the relationships involving relatedness, both directly and indirectly through achievement goals, highlighting a contextual complexity in arts education. These findings extend existing motivational theories by demonstrating differentiated mechanisms through which psychological needs influence academic performance in the arts context. Practical implications are discussed for educators and institutions seeking to support art students' motivation and academic development within Chinese higher education.

## 1 Introduction

In the context of rapid technological advancement and the growing integration of artificial intelligence, higher education systems worldwide are placing increasing

emphasis on creativity, adaptability, and innovation. In China, national strategies aimed at strengthening cultural industries and creative talent development have led to the rapid expansion of arts education at the undergraduate level. However, Chinese undergraduate students majoring in the arts (hereafter referred to as CUSMA) face a widening gap between professional skill expectations and perceived academic performance, raising concerns about their academic development and motivational sustainability (Liu, 2025). Existing educational research identifies student motivation, learning satisfaction, and psychological engagement as central determinants of academic success (Yafi et al., 2021). Recent educational quality frameworks further suggest that effective learning environments emerge from the interaction of technological support, mentorship, and community-based resources, which collectively influence students' motivation and performance (Tripon, 2025). Self-Determination Theory (SDT) highlights the importance of satisfying autonomy, competence, and relatedness for fostering intrinsic motivation and performance (Cheon and Reeve, 2015; Guay et al., 2008; Minnaert et al., 2007; Reeve and Lee, 2014; Saeki and Quirk, 2015; Skinner et al., 2008). Achievement Goal Theory further emphasizes how mastery and performance-approach goals direct motivation toward learning outcomes (Alhadabi and Karpinski, 2020; Elliot and McGregor, 2001), Together, these frameworks provide a comprehensive lens for understanding academic performance.

Despite this strong theoretical foundation, important gaps remain. Most SDT and achievement goal research has focused on general student populations, with limited attention to the distinctive pedagogical context of arts disciplines. Arts education differs from other academic fields in several critical ways: learning outcomes are often evaluated through subjective and performance-based assessments; students are frequently exposed to public critique and peer comparison; and professional identity formation is closely intertwined with emotional investment and social recognition. Empirical studies show that arts students experience higher performance anxiety, stronger dependence on teacher feedback, and greater sensitivity to social evaluation than students in many other disciplines (Sun et al., 2019). These characteristics may alter how psychological need satisfaction translates into academic performance and how contextual factors such as social support operate. Understanding these mechanisms is not merely an academic issue; it has direct implications for sustaining student motivation, reducing dropout risks, and supporting the development of creative professionals in a sector that is increasingly important for cultural and economic innovation. However, existing SDT and achievement goal research has largely been conducted in general academic contexts, with limited attention to performance-based creative disciplines where social evaluation is inherent to learning.

To address these gaps, this study constructs and tests an integrated mediation–moderation model grounded in SDT and Achievement Goal Theory within the specific context of Chinese undergraduate art majors. By examining how autonomy, competence, and relatedness influence perceived academic performance through achievement goal orientations and how these relationships are shaped by perceived social support, this research provides context-sensitive insights that extend existing motivational theories into creative disciplines. The study is original in focusing on an underrepresented yet socially significant student population and in integrating psychological needs, goal processes, and contextual resources into a unified framework. The findings are relevant for educational researchers, higher education administrators, curriculum designers, and arts educators. For these stakeholders, the study offers evidence-based guidance for creating learning environments that foster motivation while accounting for the social and evaluative pressures inherent in arts education.

## 2 Theoretical framework

Our review of the literature indicates that Self-Determination Theory (SDT) emphasizes the development of self-determined abilities (Osei and Bjorklund, 2024), Students' basic psychological needs—competence, relatedness, and autonomy—influence both internal and external motivations, which in turn affect outcomes such as academic performance. This makes SDT a suitable framework for examining the perceived academic performance of Chinese undergraduate students majoring in art (CUSMA).

### 2.1 Self-determination theory

(Deci and Ryan 1985, 2017) conceptualize Self-Determination Theory (SDT) as a theory of human motivation centered on the satisfaction of autonomy, competence, and relatedness. Within this framework, the fulfillment of these basic psychological needs facilitates optimal psychological functioning and academic engagement. Within SDT, several mini theories address motivation, development, and psychological wellbeing, with key mechanisms relating specifically to motivation and learning. Central to SDT is the fulfillment of three basic psychological needs: competence, relatedness, and autonomy. (Deci et al. 1996) argue that the fulfillment of these needs provides essential nutrients for psychological growth, integration, and flourishing. According to Gairn's elaboration, autonomy refers to the need for self-organization and behavioral regulation consistent with one's self-concept; competence refers to the need to develop abilities and interact effectively with the environment; and relatedness refers to the need to feel socially connected within the learning environment (Garn et al., 2019). SDT has been widely applied in contexts such as the workplace, commercial services, and education, and it is regarded as one of the most empirically supported motivation theories (Sun et al., 2019). In education, SDT frames learning as an exploratory process rather than the simple transmission of skills and knowledge. Students must be actively engaged in seeking self-relevant skills and knowledge by exploring evolutionarily significant tasks within their social environment, which inspires motivation to learn (Osei and Bjorklund, 2024). However, excessive demands or a lack of structure can increase cognitive load and reduce students' motivation and engagement (Evans et al., 2024). The SDT motivation continuum emphasizes the quality of motivation, which in educational settings is strongly influenced by the teacher's motivating style. Previous SDT research has primarily examined how teachers' instructional styles influence student motivation. In the present study, as outlined in the following sections, we focus on the roles of perceived social support and achievement goals.

### 2.2 Perceived academic performance

(Laguna and Escolano-Pérez 2021) define academic performance as the skills and abilities students demonstrate to evidence their knowledge across domains. Beyond objectively evaluated outcomes, academic performance also encompasses students' self-evaluations, which form the conceptual basis of the present study (Fernández-Lasarte et al., 2019). De la Fuente et al., 2008, (De la Fuente et al. 2017) conceptualize perceived academic performance (PAP) as students' cognitive evaluations of their academic outcomes, including grades and achievement-related processes. Although GPA represents an objective performance indicator, PAP captures students' subjective evaluations of their academic functioning.

A substantial body of research supports the validity of academic self-perceptions. Meta-analytic evidence indicates strong associations between self-reported and official academic records (Kuncel et al., 2005). Moreover, PAP and related academic self-perceptions have consistently shown positive associations with objective performance indicators, including GPA and school grades (Ferla et al., 2010; Respondek et al., 2017). In addition, individuals' subjective perceptions of their influence over academic outcomes have been found to significantly predict academic achievement (Stupnisky et al., 2007), further underscoring the importance of self-perceptive processes in shaping academic performance. Self-reported grades generally show high levels of accuracy, with correlations frequently exceeding 0.80 in large samples (Sticca et al., 2017). Although students show a slight tendency to over-report their performance, the overall magnitude of over- or under-reporting remains small. Longitudinal research shows that discrepancies between expected and actual achievement usually shrink as students get older and gain more feedback and experience (Pinquart and Ebeling, 2020). Domain-specific academic self-perceptions have been shown to predict GPA with considerable accuracy (Ferla et al., 2010).

From a multidimensional perspective, academic performance is shaped by cognitive, motivational, social, and educational factors (Izaguirre et al., 2023). Given the complex and context-dependent nature of academic evaluation—particularly in arts education, where assessment standards vary across institutions and disciplines and often involve qualitative judgments—perceived academic performance (PAP) provides a contextually sensitive and theoretically grounded indicator of students' academic performance within specific domains.

### 2.3 Achievement goals orientations

Attaining competence is essential for achieving academic success (Dweck, 1986; Deci et al., 1996; Nicholls, 1984). (Kayiş 2013) defines achievement goal orientations as individuals' goal-related patterns that guide learning behaviors, self-perceptions, and task-related cognitions. Two primary types are distinguished in this study:

Mastery goals are advantageous because they are associated with adaptive academic patterns that promote a deep understanding of learning tasks. Students who adopt mastery goals typically demonstrate strong academic engagement and employ effective strategies to achieve full mastery of learning tasks (Barron and Harackiewicz, 2001; Lüftenegger et al., 2017; Senko, 2019; Stavropoulou et al., 2023). Moreover, mastery-oriented students exert greater effort and display higher involvement in the learning process (Senko and Dawson, 2017).

Performance orientation emphasizes demonstrating competence and communicating success to teachers, peers, and family members. For performance-oriented students, external recognition of success is a central concern, and they allocate their efforts strategically, focusing on subjects and tasks that yield the greatest rewards (Gonzalez et al., 2001).

Research suggests that combining mastery goals with performance-approach goals is particularly beneficial for students. Mastery goals foster deep understanding and proficiency, while performance-approach goals motivate students to attain high levels of achievement. This synergistic effect has been supported by empirical studies (Barron and Harackiewicz, 2001; Schmidt et al., 2020).

### 2.4 Perceived social support

(Barrera 1986) classifies social support into three categories: social connectedness, perceived social support, and enacted social support. Among these, perceived social support refers to individuals' subjective appraisal of the availability and adequacy of support from their social network. (Cohen and Hoberman 1983) conceptualize social support as a range of resources provided through interpersonal relationships. These resources function as buffers that help individuals cope with stress and life challenges. It is a multidimensional construct, commonly comprising support from distinct sources such as family, peers (e.g., friends, classmates), and significant others in the institutional context (e.g., teachers, faculty; Zimet et al., 1988). Social support can be classified into three categories: social connectedness, perceived social support, and enacted social support. Research has further demonstrated that perceived social support plays a critical role in the developmental outcomes of children and adolescents (Demaray and Malecki, 2002). (Uchino 2004) defines social support as the comfort, care, or assistance available from others, while the American Psychological Association (2018) characterizes it as the provision of assistance or comfort to help individuals manage biological, psychological, or social stress. Perceived social support refers to an individual's cognitive appraisal of available support, which enables them to cope with trauma, stress, and anxiety. It has been shown to reduce anxiety and psychological distress while enhancing overall wellbeing (Gjesfjeld et al., 2010). Among individuals aged 15–25, higher perceived social support is associated with a greater tendency to adopt active rather than avoidant coping strategies (Mai et al., 2021). Moreover, perceptions of social support positively influence students' mental health during transitional periods in university life, including entry, adaptation to new academic years, and preparation for graduation (Cage et al., 2021). In the university setting, these dimensions play different roles: family support provides a foundational sense of security; peer support offers shared experience and belonging; and faculty support is crucial for academic guidance and validation.

### 2.5 Research model and hypotheses

**Figure 1:**
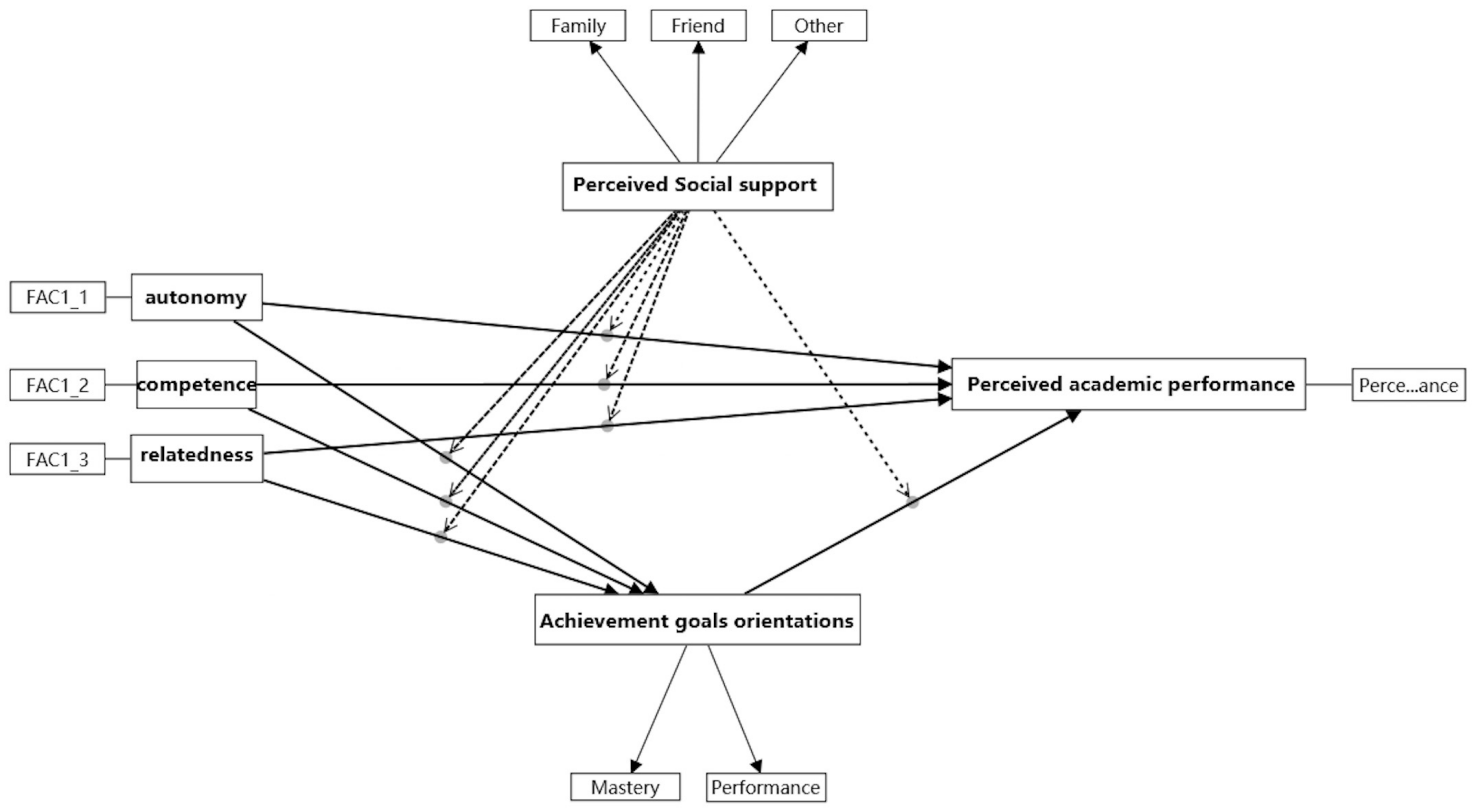
The conceptual model.

In this context, CUSMA independently manage their learning tasks, set achievement goals, and strive to accomplish them in order to improve academic performance. This process involves both intrinsic and extrinsic motivation, ultimately fostering stronger intrinsic motivation. As CUSMA improve their academic performance, their motivation increasingly becomes intrinsic. These antecedents mediate academic performance in art major learning environments through achievement goal orientation, in line with SDT. Consequently, we propose a conceptual model structured around SDT (Figure 1).

#### 2.5.1 Psychological need satisfaction and perceived academic performance

(Deci and Ryan 1985) conceptualize Self-Determination Theory as a framework in which autonomy, competence, and relatedness energize and sustain motivated behavior. When students experience autonomy, they regulate learning through personal volition, which enhances persistence and perceived academic performance. Competence strengthens students' beliefs in their capability to master academic challenges, thereby improving outcomes (Guay et al., 2008). Relatedness provides emotional security and belonging, which supports engagement, especially in socially interactive learning contexts (Reeve and Lee, 2014; Cheon and Reeve, 2015).

Arts education intensifies these mechanisms. Artistic learning involves iterative practice, critique, and public performance, requiring strong self-regulation, confidence, and social connectedness. Research shows that sustained arts training enhances attentional control and task persistence (Sun et al., 2019), suggesting that competence perceptions translate into performance. Therefore, psychological need satisfaction is expected to directly influence perceived academic performance.

**H1:** Autonomy positively predicts perceived academic performance.

**H2:** Competence positively predicts perceived academic performance.

**H3:** Relatedness positively predicts perceived academic performance.

#### 2.5.2 Mediating role of achievement goal orientations

Achievement Goal Theory explains that students' goal orientations determine how they approach learning tasks (Elliot and McGregor, 2001). Students who adopt mastery-approach and performance-approach goals invest greater effort and achieve better outcomes (Alhadabi and Karpinski, 2020; Senko and Dawson, 2017). Psychological need satisfaction shapes these goal orientations.

When students feel competent, they adopt mastery goals because they expect success (Ames, 1992; Meece et al., 2006). When students experience relatedness, social belonging encourages performance-approach goals that maintain social standing (Jiang and Zhang, 2021). Thus, competence and relatedness influence performance indirectly through achievement goal orientations.

Autonomy also contributes to self-endorsed engagement and intrinsic motivation (Sánchez-Cardona et al., 2021), which may further translate into goal pursuit. Therefore:

**H4:** Achievement goal orientations mediate the effect of autonomy on perceived academic performance.

**H5:** Achievement goal orientations mediate the effect of competence on perceived academic performance.

**H6:** Achievement goal orientations mediate the effect of relatedness on perceived academic performance.

#### 2.5.3 Moderating role of perceived social support

Perceived social support (PSS) represents a contextual resource influencing motivation (Wang et al., 2022). Support from teachers, peers, and family enhances coping capacity and emotional regulation (Gjesfjeld et al., 2010). However, in arts education, where performance is publicly evaluated and socially compared, social involvement may also function as evaluative expectation. Thus, PSS may strengthen or weaken the link between psychological needs and academic performance depending on how students interpret it. Given that social support may function as both a motivational resource and a social-evaluative pressure, it is theoretically plausible that PSS conditions the strength of both direct and indirect motivational pathways.

**H7:** Perceived social support moderates the effect of autonomy on perceived academic performance.

**H8:** Perceived social support moderates the effect of competence on perceived academic performance.

**H9:** Perceived social support moderates the effect of relatedness on perceived academic performance.

#### 2.5.4 Moderated mediation mechanism

Beyond direct effects, social support may shape how psychological needs translate into achievement goals. SDT acknowledges that social environments influence motivational internalization. In arts education, mentorship, critique, and peer comparison make social support a socio-evaluative factor influencing autonomy, competence, and relatedness simultaneously. Therefore, PSS may condition the indirect effects of psychological needs on performance through goal orientations.

**H10:** Achievement goal orientations mediate the effect of autonomy on perceived academic performance, and perceived social support moderates this mediated relationship.

**H11:** Achievement goal orientations mediate the effect of competence on perceived academic performance, and perceived social support moderates this mediated relationship.

**H12:** Achievement goal orientations mediate the effect of relatedness on perceived academic performance, and perceived social support moderates this mediated relationship (Table 1).

**Table 1:** The information of 418 students.

**Category**	**Items**	**Frequency**	**Proportion%**
Gender	Females	184	44
	Males	234	56
Local	North	106	25.35
	Central	197	47.12
	South	115	27.51
Major	Fine arts	87	20.8
	Music and dance studies	95	22.7
	Drama and film studies	79	18.9
	Art theory	64	15.3
	Design studies	93	22.2
Grade	1	88	21.1
	2	184	44
	3	109	26.1

## 3 Methodologies

### 3.1 Participants

This study was conducted within the context of Chinese higher education, focusing on undergraduate students majoring in the arts, a population experiencing rapid expansion due to national strategies promoting cultural industries and creative innovation. Arts programs in Chinese universities emphasize performance-based assessment, studio practice, and continuous evaluative feedback, making art students particularly sensitive to motivational processes and perceived academic performance; therefore, this group represents a meaningful context for examining motivational mechanisms grounded in Self-Determination Theory (SDT). A quantitative cross-sectional survey design was adopted to examine structural relationships among psychological constructs and test mediation–moderation models at a single point in time. The target population consisted of undergraduate students enrolled in arts-related majors, and the unit of analysis was the individual student, selected because arts education involves creativity, subjective evaluation, public performance, and strong social comparison that may shape motivational processes differently from other disciplines. Participants were recruited using a convenience sampling strategy through widely used student platforms in China (WENJUANXING, WeChat, QQ, and XIAOHONGSHU), enabling access to art students from Central, North, and South China. Inclusion criteria required participants to be undergraduate art majors in good physical and mental health who provided informed consent, while exclusion criteria removed incomplete responses, duplicate IP submissions, and questionnaires completed in unrealistically short times. The main study variables included psychological need satisfaction (autonomy, competence, and relatedness), achievement goal orientations, perceived social support, and perceived academic performance, all measured using previously validated instruments adapted to the arts learning context. Psychological needs were assessed using established autonomy, competence, and relatedness subscales; achievement goal orientations were measured using the Achievement Goal Questionnaire–Revised (AGQ-R); perceived social support was measured with the Multidimensional Scale of Perceived Social Support (MSPSS); and perceived academic performance was measured using the academic performance subscale of the Brief School Adjustment Scale. Data were collected via an online questionnaire administered through WENJUANXING over a 21-day period (June 25–July 15), with voluntary and anonymous participation, and a pilot test with 50 students was conducted beforehand to ensure clarity and relevance of items. Data were analyzed using SPSS 26.0, AMOS 24, and SmartPLS 4.1.1.1; preliminary analyses included descriptive statistics and reliability assessment using Cronbach's alpha and composite reliability, followed by Confirmatory Factor Analysis (CFA) to evaluate the measurement model through factor loadings, average variance extracted (AVE), and model fit indices (χ^2^/df, RMSEA, CFI, TLI, SRMR). Subsequently, Partial Least Squares Structural Equation Modeling (PLS-SEM) was employed to test the hypothesized direct, mediating, and moderating relationships among latent constructs, with bootstrapping (5,000 resamples) used to assess the significance of path coefficients.

#### 3.1.1 Need satisfaction

To assess the degree to which participants experienced the satisfaction of the three basic psychological needs (competence, relatedness, and autonomy), three previously validated questionnaires were employed. Scores from these subscales served as indicators of the latent construct.

Perceived competence. perceived competence in art-related learning was assessed using five items from the competence subscale of the Intrinsic Motivation Inventory (Mcauley et al., 1998). The IMI has been widely applied in various contexts, including reading, learning, writing, and puzzle-solving tasks. In the present study, an additional reverse-scored item was included (e.g., “I can't do Art learning class very well”). Participants responded to items such as “I think I am pretty good” and “I am satisfied with my performance,” preceded by the stem “In this art learning context…”. Responses were made on a seven-point Likert scale ranging from 1 (strongly disagree) to 7 (strongly agree). Previous research has demonstrated adequate reliability of the five-item version in undergraduate physical education contexts.

Relatedness. Relatedness was measured using the acceptance subscale of the Need for Relatedness Scale (Richer and Vallerand, 1998). Originally developed for workplace settings, the stem was adapted in the present study to reflect the academic context: “With the other students in this art learning context I feel…”. Items included “close,” “valued,” and “supported.” Responses were recorded on a seven-point Likert scale ranging from 1 (strongly disagree) to 7 (strongly agree). Prior research with students in higher education contexts has provided evidence for the reliability of this measure (Galindo-Domínguez et al., 2025).

Autonomy. Autonomy was assessed using five items adapted from (Standage et al. 2003), which were originally collated from research on perceptions of autonomy in physical education and other life domains (Blais et al., 1990; Ntoumanis, 2001). One reverse-scored item was added (“I have to force myself to do the activities”). Example items included “I have some choice in what I want to do” and “I have a say regarding what skills I want to practice,” preceded by the stem “In this art learning context…”. Responses were made on a seven-point Likert scale (1 = strongly disagree, 7 = strongly agree). Previous studies with secondary school students have confirmed the internal reliability of this five-item scale (Standage et al., 2003).

#### 3.1.2 Perceived social support

Perceived social support was measured using the Multidimensional Scale of Perceived Social Support (Zimet et al., 1988). The MSPSS is a 12-item instrument designed to assess subjective perceptions of the adequacy of social support among university students. Participants responded on a seven-point Likert scale ranging from 1 (very strongly disagree) to 7 (very strongly agree). The scale captures support from three sources: family, friends, and significant others. Item scores were summed to compute a total score, with higher scores reflecting greater perceived social support. Previous studies have demonstrated the internal reliability of the 12-item MSPSS in university student populations, including research on perceived social support and suicidal thoughts among young adults (Howlader et al., 2024). The total score of the MSPSS was used to represent the overall level of perceived social support.

#### 3.1.3 Perceived academic performance

Perceived academic performance (PAP) was used as the primary outcome measure in this study. Since art programs across Chinese universities lack a unified grading system, PAP—a subjective, self-reported measure—allows for consistent assessment of students' own appraisal of their academic progress. This approach also aligns with the study's focus on psychological and motivational processes. Perceived academic performance was measured using the Escala Breve de Ajuste Escolar [Brief School Adjustment Scale] (Rubia et al., 2010). The full questionnaire consists of 10 items rated on a six-point Likert scale (1 = completely disagree, 6 = completely agree) and assesses three domains: academic expectations (2 items), academic performance (3 items), and school integration problems (5 items). For the present study, only the academic performance subscale was employed, which evaluates students' perceptions of their performance as learners. Prior research has supported the internal reliability of this 3-item subscale in studies of secondary school students' perceived academic performance (Izaguirre et al., 2023).

#### 3.1.4 Achievement goals orientations

Achievement goal orientations were measured using the Achievement Goal Questionnaire–Revised (AGQ-R) combined with selected items from the original Achievement Goal Questionnaire (AGQ). The instrument includes 12 items rated on a five-point Likert scale (1 = strongly disagree, 5 = strongly agree). For the purposes of this study, only the mastery-approach goal (3 items) and performance-approach goal (3 items) subscales were utilized. Previous research has provided support for the internal reliability of this 6-item version in assessing undergraduates' achievement goal orientations (Lee, 2023).

## 4 Results

### 4.1 Descriptive statistics

The final sample consisted of 418 undergraduate art majors after data cleaning procedures removed invalid responses, yielding a valid response rate of 92.07%. Participants included 184 females and 234 males, with a mean age of 22.8 years (SD = 2.31). Students were recruited from multiple universities across China, with the largest proportion from Central China (*n* = 197), followed by North China (*n* = 106) and South China (*n* = 115). According to the Catalog of Undergraduate Specialties in General Higher Education issued by the Ministry of Education of China, participants represented five art-related specialties: Fine Arts (*n* = 87), Music and Dance Studies (*n* = 95), Drama and Film Studies (*n* = 79), Art Theory (*n* = 64), and Design Studies (*n* = 93). The sample also covered all academic years, including first-year (21.1%), second-year (44.0%), third-year (26.1%), and fourth-year students (8.9%).

### 4.2 Measurement model results

**Figure 2:**
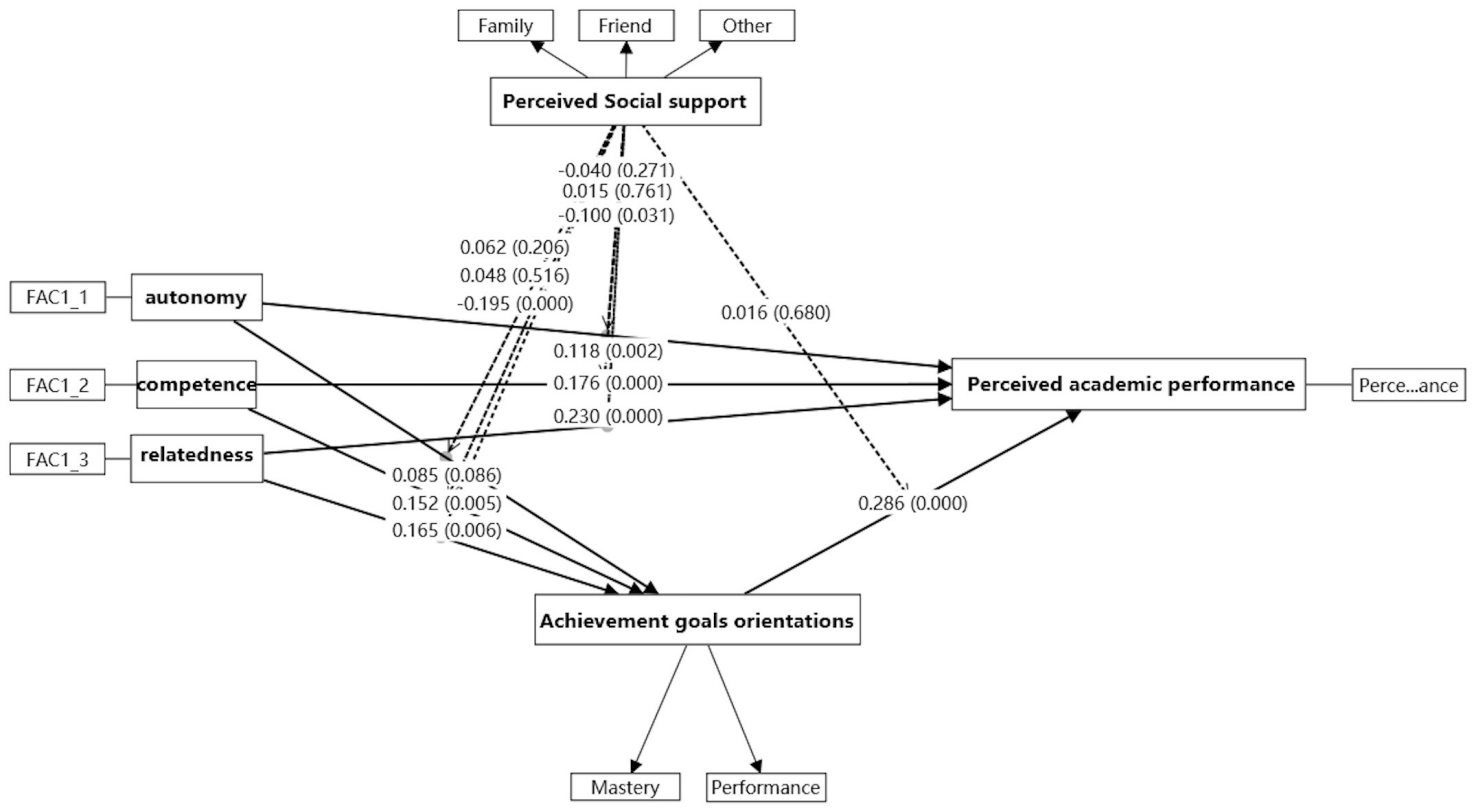
SEM results obtained using Smart PLS.

Table 2 presents the descriptive statistics for the nine factors. The mean values ranged from 3.636 to 5.129, with standard deviations (SD) between 0.584 and 1.262. Pairwise correlations were examined using Pearson's correlation test, and the coefficients are reported in Table 2. All pairwise correlations were significant at the 0.01 level (*p* < 0.01). Among them, the smallest correlation was observed between Achievement Goal Performance (AGP) and Autonomy (A) (*r* = 0.463, *p* < 0.01), while the strongest correlation was observed between relatedness and perceived academic performance (*r* = 0.698, *p* < 0.01). Overall, the nine factors in the proposed model demonstrated significant and positive intercorrelations, supporting their theoretical associations. Confirmatory Factor Analysis (CFA) was conducted to test and validate the hypothetical factor structure of the measurement instruments. CFA is a statistical technique used to determine whether the relationships among observed variables are consistent with the factor structure proposed a priori (Brown, 2015; NoAli et al., 2019). The analysis involved specifying the measurement model, estimating factor loadings, and evaluating overall model fit. Consistent with established guidelines, factor loadings ≥ 0.70 were considered satisfactory (Black et al., 2010). As shown in Table 3, all standardized factor loadings exceeded 0.83, supporting the construct validity of the measurement model. Model fit indices are reported in Table 4 (Line 2) and depicted in Figure 2. The chi-square to degrees of freedom ratio (χ^2^/df) was below the recommended threshold of 3, indicating good fit. The root means square error of approximation (RMSEA = 0.049) and the standardized root mean square residual (SRMR = 0.0316) were below the recommended cutoff of 0.05, further supporting model adequacy. In addition, the Comparative Fit Index (CFI = 0.958), Tucker–Lewis Index (TLI = 0.953), and Incremental Fit Index (IFI = 0.959) all surpassed the recommended threshold of 0.90. Although the Goodness-of-Fit Index (GFI = 0.87) and the Adjusted Goodness-of-Fit Index (AGFI = 0.845) were slightly lower than the ideal value of 0.90, they exceeded the minimum acceptable cutoff of 0.80. Taken together, these results indicate that the nine-factor model demonstrates a strong and acceptable fit, thereby confirming the hypothesized measurement structure.

**Table 2:** The preliminary analysis results of 418 students.

**Variable**	**M**	**SD**	**A**	**C**	**R**	**PAP**	**AGM**	**AGP**	**O**	**F**	**D**
A	4.8	1.06595	0.848								
C	4.6463	1.09523	0.696^**^	0.867							
R	4.9089	1.01411	0.662^**^	0.698^**^	0.87						
PAP	3.636	0.70758	0.648^**^	0.688^**^	0.698^**^	0.883					
AGM	3.8167	0.65916	0.600^**^	0.551^**^	0.619^**^	0.698^**^	0.873				
AGP	3.7325	0.72121	0.463^**^	0.560^**^	0.530^**^	0.635^**^	0.684^**^	0.883			
O	4.8237	1.29637	0.464^**^	0.501^**^	0.519^**^	0.543^**^	0.520^**^	0.527^**^	0.908		
F	5.0671	1.2966	0.522^**^	0.509^**^	0.567^**^	0.602^**^	0.601^**^	0.533^**^	0.612^**^	0.904	
D	5.1289	1.17013	0.554^**^	0.553^**^	0.672^**^	0.657^**^	0.620^**^	0.581^**^	0.688^**^	0.747^**^	0.902

A, Autonomy; C, Competence; R, Relatedness; PAP, Perceived academic performance; AGM, Mastery.

AGP, Performance; O, Other support; F, Family support D, Friend support; **p < 0.01.

**Table 3:** SEM verification results of the proposed model.

**Construct**	**Item**	**Factor loadings**	**SMC**	**Ave**	**α**	**CR**
Mastery	AGM01	0.873	0.678	0.762	0.844	0.906
	AGM02	0.86	0.642			
	AGM03	0.888	0.607			
Performance	AGP01	0.897	0.693	0.78	0.859	0.914
	AGP02	0.887	0.722			
	AGP03	0.867	0.602			
Perceived academic performance	PAP01	0.885	0.674	0.782	0.86	0.915
	PAP02	0.873	0.68			
	PAP03	0.895	0.666			
Friends	PSD01	0.899	0.771	0.815	0.924	0.946
	PSD02	0.911	0.761			
	PSD03	0.919	0.786			
	PSD04	0.883	0.701			
Family	PSF01	0.912	0.808	0.819	0.926	0.947
	PSF02	0.918	0.802			
	PSF03	0.921	0.777			
	PSF04	0.868	0.653			
Others	PSO01	0.906	0.753	0.825	0.929	0.95
	PSO02	0.919	0.816			
	PSO03	0.92	0.791			
	PSO04	0.889	0.712			
Autonomy	SA01	0.869	0.68	0.719	0.902	0.927
	SA02	0.832	0.631			
	SA03	0.837	0.662			
	SA04	0.869	0.687			
	SA05	0.836	0.6			
Competence	SC01	0.876	0.71	0.752	0.917	0.938
	SC02	0.87	0.719			
	SC03	0.852	0.661			
	SC04	0.903	0.77			
	SC05	0.835	0.61			
Relatedness	SR01	0.864	0.681	0.757	0.92	0.94
	SR02	0.882	0.716			
	SR03	0.869	0.694			
	SR04	0.871	0.703			
	SR05	0.864	0.693			

**Table 4:** The model fit of different number of factors.

**Model**	**CMIN**	**DF**	**CMIN/DF**	**RMSEA**	**SRMR**	**GFI**	**AGFI**	**CFI**	**TLI**	**IFI**
Threshold values			< 3	< 0.05	< 0.05	no < 0.85	no < 0.80	no < 0.9	no < 0.9	no < 0.9
	1,113.891	558	1.996	0.049	0.0316	0.87	0.845	0.958	0.953	0.959

Construct validity is a critical aspect of psychological and educational measurement and consists of two key components: convergent validity (CV) and discriminant validity (DV; Campbell-Sills and Stein, 2007). Establishing both is essential to confirm that an instrument measures its intended construct while remaining distinct from related but different constructs. Convergent validity was assessed using the Average Variance Extracted (AVE), with a threshold value of ≥ 0.50 considered satisfactory (Hair et al., 2010). As shown in Table 3, all AVE values exceeded the cutoff, demonstrating adequate CV. Discriminant validity was evaluated using the Fornell–Larcker criterion, which requires the square root of the AVE for each construct to be greater than its correlations with other constructs (Fornell and Larcker, 1981). As reported in Table 2, this condition was met across all constructs, confirming satisfactory DV. Taken together with the model fit indices in Table 4, the findings provide strong evidence that the proposed model demonstrates acceptable construct validity.

To further evaluate measurement quality, construct reliability (CR) and Cronbach's α were examined as indicators of internal consistency. Both indices should ideally exceed 0.70 (Ware et al., 1996; Bagozzi and Yi, 1989). In this study, CR values ranged from 0.906 to 0.950, while Cronbach's α ranged from 0.844 to 0.929, well above the recommended threshold. These results confirm that the proposed model demonstrates excellent internal consistency and reliability.

### 4.3 Structural model results

Structural equation modeling results indicated that eight of the 14 hypothesized paths were statistically supported (see Table 5). Following (Hayes 2013), bias-corrected bootstrapping with 5,000 resamples was used, and effects were considered significant when the 95% confidence interval did not include zero (see Table 6). Several direct effects reached significance at the 0.01 level, while additional paths demonstrated strong statistical significance (*p* < 0.001). The remaining paths did not reach statistical significance. These findings provide partial support for the proposed model and indicate that both direct and indirect mechanisms operate differently across psychological needs.

**Table 5:** The path direct effect.

**No**.	**Path of action**	**β**	**SD**	***T***	***P***	**0.025**	**0.975**	**Results**
1	A -> PAP	0.118	0.038	3.136	0.002	0.047	0.194	Accepted
2	C -> PAP	0.176	0.049	3.562	***	0.085	0.274	Accepted
3	R -> PAP	0.23	0.048	4.805	***	0.132	0.319	Accepted
4	AGO -> PAP	0.286	0.05	5.722	***	0.185	0.383	Accepted
5	A -> AGO	0.085	0.049	1.72	0.086	−0.011	0.182	Rejected
6	C ->AGO	0.152	0.054	2.819	0.005	0.05	0.257	Accepted
7	R -> AGO	0.165	0.06	2.765	0.006	0.044	0.278	Accepted
8	PSS x A -> PAP	−0.04	0.036	1.1	0.271	−0.111	0.031	Rejected
9	PSS x C -> PAP	0.015	0.051	0.305	0.761	−0.086	0.114	Rejected
10	PSS x R -> PAP	−0.1	0.046	2.162	0.031	−0.188	−0.003	Accepted
11	PSS x AGO -> PAP	0.016	0.04	0.412	0.68	−0.067	0.089	Rejected
12	PSS x A -> AGO	0.062	0.049	1.265	0.206	−0.037	0.159	Rejected
13	PSS x C -> AGO	0.048	0.073	0.65	0.516	−0.097	0.191	Rejected
14	PSS x R -> AGO	−0.195	0.049	3.958	***	−0.288	−0.092	Accepted

(^***^p < 0.001).

**Table 6:** Standardized bootstrap mediation effect test.

**Hypothesis**	**Path**	**β**	**SD**	***T***	***P***	**0.025**	**0.975**	**Results**
H1	A -> PAP	0.118	0.038	3.136	0.002	0.047	0.194	Accepted
H2	C -> PAP	0.176	0.049	3.562	***	0.085	0.274	Accepted
H3	R -> PAP	0.23	0.048	4.805	***	0.132	0.319	Accepted
H4	A -> AGO -> PAP	0.024	0.014	1.707	0.088	−0.003	0.053	Rejected
H5	C ->AGO -> PAP	0.043	0.017	2.497	0.013	0.013	0.081	Accepted
H6	R ->AGO -> PAP	0.047	0.019	2.44	0.015	0.011	0.087	Accepted
H7	PSS x A -> PAP	−0.04	0.036	1.1	0.271	−0.11	0.031	Rejected
H8	PSS x C -> PAP	0.015	0.051	0.305	0.761	−0.09	0.114	Rejected
H9	PSS x R -> PAP	−0.1	0.046	2.162	0.031	−0.19	−0.003	Accepted
H10	PSS x A -> AGO -> PAP	0.018	0.014	1.238	0.216	−0.01	0.047	Rejected
H11	PSS x C -> AGO -> PAP	0.014	0.021	0.652	0.514	−0.03	0.054	Rejected
H12	PSS x R -> AGO -> PAP	−0.056	0.016	3.466	0.001	−0.09	−0.024	Accepted

(^***^p < 0.001).

Bootstrapping procedures following (Hayes, 2013) were employed with 5,000 resamples and 95% bias-corrected confidence intervals (CI) to assess indirect and moderated effects (see Table 6). An effect was considered statistically significant when the confidence interval did not include zero.

The indirect effect of autonomy on perceived academic performance via achievement goal orientations (A → AGO → PAP) was not significant (β = 0.024, SD = 0.014, *p* = 0.088), as the confidence interval included zero. In contrast, the indirect effects of competence (C → AGO → PAP: β = 0.043, SD = 0.017, *p* = 0.013) and relatedness (R → AGO → PAP: β = 0.047, SD = 0.019, *p* = 0.015) were significant, with confidence intervals not crossing zero. These findings indicate that achievement goal orientations mediate the effects of competence and relatedness, but not autonomy.

Regarding moderated mediation, the interaction effects involving perceived social support (PSS) showed differentiated patterns. The conditional indirect effects of PSS × A → AGO → PAP (β = 0.018, *p* = 0.216) and PSS × C → AGO → PAP (β = 0.014, *p* = 0.514) were not significant, as their confidence intervals included zero. However, the moderated mediation pathway for relatedness (PSS × R → AGO → PAP) was significant (β = −0.056, SD = 0.016, *p* = 0.001), with a confidence interval excluding zero, indicating that perceived social support negatively conditions the indirect effect of relatedness on performance through achievement goal orientations.

For direct moderation, the interaction effects of PSS with autonomy (PSS × A → PAP: β = −0.040, *p* = 0.271) and competence (PSS × C → PAP: β = 0.014, *p* = 0.761) were not significant. In contrast, PSS significantly moderated the relatedness–performance link (PSS × R → PAP: β = −0.100, SD = 0.046, *p* = 0.031), with a confidence interval not including zero, indicating a negative moderation effect.

## 5 Discussion

### 5.1 Purpose and contributions

This study examined the motivational mechanisms underlying perceived academic performance (PAP) among Chinese undergraduate art majors within the frameworks of Self-Determination Theory (SDT) and Achievement Goal Theory, and the findings further support the construct validity of PAP as a meaningful outcome indicator in higher education research. By integrating psychological need satisfaction, achievement goal orientations (AGO), and perceived social support (PSS) into a unified mediation–moderation model, this research extends SDT to the distinctive pedagogical context of arts higher education. Unlike many prior studies focusing on general student populations, the present study demonstrates that motivational processes operate differently across autonomy, competence, and relatedness in creative disciplines, thereby addressing an important gap in understanding the academic development of Chinese art majors. Thus, this study extends SDT by demonstrating that the functional role of psychological needs varies across disciplinary contexts and that social support may operate as both a motivational resource and an evaluative constraint.

### 5.2 Results to hypotheses

The present study aimed to clarify how basic psychological need satisfaction influences perceived academic performance among Chinese undergraduate art majors, as well as to uncover the motivational and contextual mechanisms underlying these relationships. The findings largely support the core assumptions of Self-Determination Theory (SDT), while also revealing discipline-specific motivational dynamics.

First, the significant direct effects of autonomy, competence, and relatedness on perceived academic performance (H1–H3) reaffirm SDT's central proposition that the fulfillment of basic psychological needs constitutes a fundamental driver of adaptive functioning and performance. In the context of arts education, where learning processes involve creativity, performance evaluation, and public presentation, the satisfaction of these needs appears to play an especially salient role in sustaining students' engagement and self-perceived achievement. These findings align with prior SDT research demonstrating that need satisfaction promotes academic outcomes across educational settings (Cheon and Reeve, 2015; Guay et al., 2008; Reeve and Lee, 2014), but extend this evidence to performance-based creative disciplines, where evaluation criteria are often subjective and socially visible.

Second, the mediation analysis revealed differentiated motivational pathways. Achievement goal orientations mediated the effects of competence and relatedness on academic performance (H5–H6), but not autonomy (H4). In creative disciplines, autonomy may operate as intrinsic regulatory engagement rather than structured goal pursuit, which explains why its effect does not depend on achievement goal orientations. This pattern suggests that feeling capable and socially connected may encourage students to translate their motivational resources into goal-directed engagement, consistent with Achievement Goal Theory's view that mastery and performance-approach goals organize effort and persistence (Elliot and McGregor, 2001; Ames, 1992). In contrast, autonomy's influence appears to operate more directly. In creative disciplines, autonomy may function less as a precursor to structured goal setting and more as an intrinsic regulatory state, characterized by self-endorsed engagement and personal meaning. This interpretation is consistent with SDT's conceptualization of autonomy as a quality of motivational regulation rather than merely a situational antecedent to goal adoption.

Third, the moderating effects of perceived social support (PSS) revealed a more complex picture. PSS did not significantly moderate the autonomy–performance or competence–performance relationships (H7–H8), suggesting that these needs may exert relatively stable influences across varying levels of perceived support. However, PSS negatively moderated the relationship between relatedness and academic performance (H9) and weakened the indirect pathway via achievement goal orientations (H12). This finding aligns with SDT's distinction between autonomy-supportive and controlling environments, suggesting that support accompanied by performance expectations may shift motivational regulation from self-determined to socially evaluated forms. This finding underscores that the functional meaning of social support is culturally and contextually embedded rather than universally facilitative. From an SDT perspective, social environments can shift from autonomy-supportive to controlling when support is accompanied by implicit expectations, social comparison, and performance pressure (Deci and Ryan, 2000). In Chinese arts education, where learning is often intertwined with teacher mentorship, public critique, and familial expectations, high levels of perceived support may carry evaluative components that intensify performance pressure. Under such conditions, the motivational benefits of relatedness may be attenuated, and goal orientations may become more externally regulated. This interpretation helps explain the counterintuitive negative moderation and situates the findings within SDT's broader distinction between supportive and controlling social contexts.

Overall, these results extend SDT by demonstrating that the functional role of psychological needs is not uniform across disciplines. In performance-evaluative creative domains, autonomy may operate primarily through direct intrinsic regulation, while competence and relatedness may depend more on goal-structured motivational processes. Moreover, social support may simultaneously serve as a motivational resource and an evaluative constraint, highlighting the contextual complexity of support systems in higher education.

### 5.3 Conclusion and implications

This study refines SDT applications by demonstrating that the three psychological needs do not operate uniformly in creative disciplines. Autonomy primarily influences academic performance through direct motivational pathways, whereas competence and relatedness partly rely on achievement goal processes. Furthermore, the negative moderating role of PSS challenges the common assumption that social support uniformly enhances motivation, suggesting instead that its effects depend on evaluative and cultural contexts. These findings contribute to a more nuanced understanding of how social environments interact with motivational mechanisms in performance-based learning domains.

### 5.4 Practical implications

The findings suggest several implications for arts education. Educators should foster autonomy-supportive environments that encourage independent creative exploration while strengthening students' sense of competence through constructive feedback and scaffolded skill development. Relatedness can be promoted through collaborative learning and supportive peer interaction; however, social support should be structured to minimize evaluative pressure. In the Chinese cultural context, where mentorship traditions and family expectations are strong, support may sometimes be perceived as obligation rather than encouragement. Therefore, teachers and institutions should balance guidance with students' autonomy and goal ownership. Diverse assessment methods aligned with arts pedagogy may further reduce excessive performance pressure.

### 5.5 Limitations and future research

Several limitations warrant consideration. First, the cross-sectional design limits causal inference; longitudinal studies are needed to examine motivational dynamics over time. Second, the sample was drawn from Chinese universities, which may limit generalizability; future research should include broader and cross-cultural samples. Third, additional contextual and personal variables—such as family background, cultural values, and personality traits—should be incorporated to better capture the complexity of motivational processes. Finally, future studies could explore reciprocal relationships, as academic performance may in turn influence psychological needs and goal orientations.

## Data Availability

The datasets presented in this study can be found in online repositories. The names of the repository/repositories and accession number(s) can be found in the article/supplementary material.
